# Fuels: Corn Ethanol Goal Revives Dead Zone Concerns

**DOI:** 10.1289/ehp.116-a242

**Published:** 2008-06

**Authors:** Carol Potera

The Energy Independence and Security Act of 2007 calls for the production of 36 billion gallons of renewable fuels by 2022, including 15 billion gallons of corn-based ethanol, a tripling of current production that would require a similar increase in corn production. Yet scientists are coming to understand that biofuels, which originally sounded like a sensible response to the twin problems of climate change and dependence on foreign oil, create environmental problems of their own. One such problem is an increase in nitrogen runoff as farmers rush to plant more corn to meet growing demand for ethanol. According to the National Corn Growers Association, rising corn prices prompted farmers to plant 92.9 million acres of the grain in 2007, a 19% increase over the prior year.

Fred Below, a professor of crop physiology at the University of Illinois at Urbana–Champaign, explains that corn requires more nitrogen fertilizer compared with other crops because of its higher production of grain per unit area than other crops. “Also,” he adds, “unlike crops like soybeans that form symbiotic relationships with soil bacteria to obtain a portion of their nitrogen from the atmosphere, corn is completely dependent on available nitrogen in soil.” Naturally occurring nitrogen usually must be supplemented with fertilizer to meet corn’s needs.

The nitrogen applied as fertilizer to corn does not stay in the Corn Belt. Instead, it travels via local streams and rivers to the Mississippi River and eventually enters the Gulf of Mexico. Once there, the excess nitrogen fuels explosive algal blooms. When the algae die, they are decomposed by bacteria that consume much of the oxygen in the water. The result is a so-called Dead Zone about the size of New Jersey that is so depleted of oxygen that fish, shellfish, and other aquatic life cannot survive there. The boom in biofuel production “is a disaster for the Gulf of Mexico,” says Simon Donner, an assistant geography professor at the University of British Columbia. “Nitrogen already is a big problem, and the new energy policy will make it worse.”

Donner and Chris Kucharik, an associate scientist at the University of Wisconsin–Madison, are the first to quantify how the corn ethanol boom may impact the Gulf. They used established models that combine agricultural land use with nitrogen cycling. The results, reported 18 March 2008 in *Proceedings of the National Academy of Sciences*, showed that scaling up corn production to meet the 15-billion-gallon goal would increase nitrogen loading in the Dead Zone by 10–18%. This would boost nitrogen levels to twice the level recommended by the Mississippi Basin/Gulf of Mexico Water Nutrient Task Force, a coalition of federal, state, and tribal agencies that has monitored the Dead Zone since 1997. The task force says a 30% reduction of nitrogen runoff is needed if the Dead Zone is to shrink.

Streams serve as natural filters to prevent nitrate pollution from reaching coastal waters. Bacteria in stream sediments remove nitrogen through denitrification, a process that converts nitrate to benign nitrogen gases that diffuse from the water into the air. However, increased levels of nitrogen runoff in local streams from urban and agriculture land use can overwhelm streams, which “become very inefficient and do not perform the ecological service we assume they do,” says Patrick Mulholland, an aquatic ecologist at Oak Ridge National Laboratory.

Mulholland coordinated a team of 31 ecologists who monitored 72 streams in 8 regions of the United States. The streams received runoff from agricultural lands, urban areas such as golf courses and housing subdivisions, or wild-growth vegetation. The team injected a small amount of ^15^N, a safe isotope of nitrogen, into waterways to track nitrogen movement and removal as small streams flowed into larger ones.

Although denitrification rates increased in tandem with rising nitrate concentrations, the process became very inefficient with a much smaller proportion of the nitrate removed from stream waters at higher nitrate concentrations. “Humans can easily overload stream and river networks, so a smaller proportion of the nitrate load is removed from the entire system,” says Mulholland. The team published these results in the 13 March 2008 issue of *Nature*.

Many scientists suggest converting cornfields to feedstocks that need little or no fertilization, such as switchgrass. No longer a dubious prospect, switchgrass proved its economic worth in the first large-scale field trial of the crop, published in the 15 January 2008 *Proceedings of the National Academy of Sciences*. Ten farmers in Nebraska and the Dakotas grew 15- to 20-acre plots of switch-grass and recorded fuel, fertilizer, and herbicide inputs and grass production over a 5-year period. Plant geneticist Kenneth Vogel and colleagues at the U.S. Department of Agriculture (USDA) Agricultural Research Service at the University of Nebraska–Lincoln plugged these numbers into a biofuel analysis “meta-model” developed at the University of California, Berkeley, and described in the 27 January 2006 issue of *Science*. They calculated that the 3- to 5-foot-tall grass generated 540% more renewable energy than was needed to grow, harvest, and process it into ethanol. Moreover, switchgrass produced an average biomass equivalent of 320 gallons of ethanol per acre, similar to regional yields from corn. What’s more, they found that ethanol made from switchgrass emitted 94% less greenhouse gases compared with burning gasoline.

The baseline study “clearly demonstrates that switchgrass grown for biomass energy is very net energy positive, and its potential as a biomass energy crop on marginal cropland is promising,” Vogel says. Moreover, switch-grass residue remaining after ethanol processing could fuel biorefineries, whereas corn biorefineries burn natural gas or other fossil fuels. Vogel says USDA researchers are breeding new switchgrass cultivars specifically for bioenergy that grow to over 8 feet tall, yielding more biomass per acre.

The trick to advancing the use of alternative feedstocks such as switchgrass, corn-stalks, and wood waste lies in finding enzymes to degrade the xylan, cellulose, and lignin in these cellulosic materials. Collectively known as lignocellulose, these three components make these plants strong and recalcitrant to known enzymatic methods of degradation.

Termites, however, rapidly digest wood. These insects have detailed mechanisms for converting lignocellulose into their own biofuel, says study coordinator Jared Leadbetter, an associate professor of environmental microbiology at the California Institute of Technology. A public–private team has now sequenced microorganisms that live in the hindgut of *Nasutitermes* termites to identify how the termites achieve this feat.

Two types of bacteria dominate the termite hindgut—treponomes, which ferment sugar, and fibrobacters, which specialize in breaking down lignocellulose. Several hundred genes related to enzymatic digestion of lignocellulose were identified. The team, which reported these results in the 22 November 2007 issue of *Nature*, is now growing the microbes in the laboratory in an attempt to better characterize the enzymes and pathways relevant for lignocellulose degradation.

Bacteria can generate another alternative fuel—hydrogen, a renewable, efficient, and clean energy source that powers fuel cells. *Escherichia coli*, better known for causing food poisoning, produces hydrogen when it feeds on glucose and formate, common ingredients in waste streams from sugar beet processing plants and breweries. In a bid to harness this potential fuel source, Thomas Wood, a chemical engineer at Texas A&M University, revved up hydrogen yields by inducing mutations in key metabolic pathways. A modified *E. coli* strain that feeds on glucose was shown to produce 5 times more hydrogen than the parent strain, and a formate-eating strain produced 141 times more hydrogen, as described in the January 2008 issue of *Microbial Biotechnology*.

Whereas ethanol production converts biomass into sugars, “we want to take that sugar and turn it into hydrogen,” says Wood. He envisions adding mixtures of microbes to waste streams that could devour different sugars and generate hydrogen. Ultimately, he says, homes may be outfitted with 250-gallon bioreactors (about the size of a home heating oil tank) to convert sugary wastes into hydrogen onsite to power appliances, lights, and computers. “It’s an exciting time to be involved in developing different fuel sources,” Wood says, “but we have to do it in a way that does not hurt the environment.”

## Figures and Tables

**Figure:**
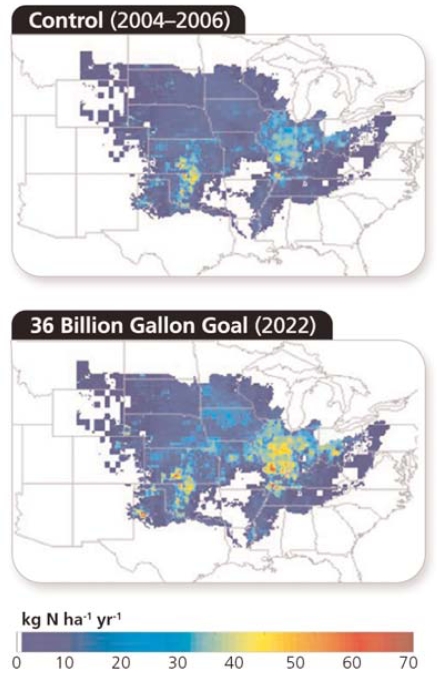
Simulated nitrogen loading reflects corn production in 2004–2006 and in 2022 if the United States were to achieve the goal of producing 36 billion gallons of renewable fuels (including 15 billion gallons of corn ethanol) set forth in the Energy Independence and Security Act. **Source:** Donner and Kucharik. Proc Natl Acad Sci USA 105:4513–4518 (2008).

**Figure:**
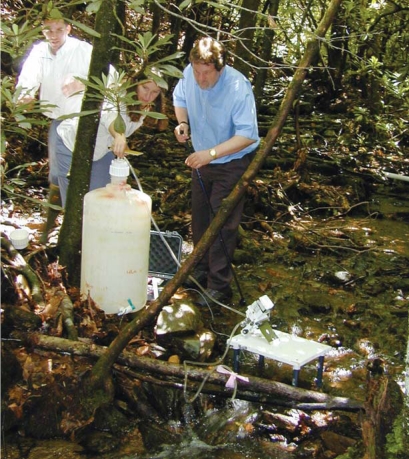
Adding nitrogen to stream waters (as here, in the Coweeta Experimental Forest of western North Carolina) allowed researchers to study how agricultural runoff might wend its way from cornfields to the Gulf of Mexico.

